# Supplementary material for: Reconciling Mining with the Conservation of Cave Biodiversity: A Quantitative Baseline to Help Establish Conservation Priorities
Source: PLoS One. 2016 Dec 20;11(12):e0168348. doi: 10.1371/journal.pone.0168348 (PMC5173368; doi:10.1371/journal.pone.0168348)
Supplement: S1 Dataset — (ZIP) [file pone.0168348.s002.zip › Taxa/Serra Norte/SN_2013/N5S/Lista N5S-40.pdf]

CAVIDADE N5S-0040

Classe	Ordem	Fam/Outros	Gên/Outros	Espécie	S-U	U-S	Zon.
Arachnida	Acari	Rhagidiidae		sp.3	X		E
Arachnida	Araneae	Araneidae	*Alpaida*	aff. *negro*	X	X	E
Arachnida	Araneae	Drymusidae		jovem	X	X	E
Arachnida	Araneae	Mysmenidae	*Microdipoena*	sp.1		X	E
Arachnida	Araneae	Ochyroceratidae	*Ochyrocera*	sp.1		X	E
Arachnida	Araneae	Ochyroceratidae	*Ochyrocera*	sp.3	X		E
Arachnida	Araneae	Oonopidae		jovem	X		E
Arachnida	Araneae	Pholcidae	Ninetinae	sp.1	X		E
Arachnida	Araneae	Salticidae		sp.13	X	X	E
Arachnida	Araneae	Scytodidae	*Scytodes*	jovem	1		E
Arachnida	Araneae	Theridiidae	*Theridion*	sp.1		X	E
Arachnida	Araneae	Theridiosomatidae	*Plato*	sp.1	X	X	E
Chilopoda	Scutigeromorpha	Pselliodidae	*Sphendononema*	jovem		2	E
Entognatha	Collembola	Paronellidae		sp.1	X	X	E
Entognatha	Diplura	Campodeidae		sp.1		X	E
Insecta	Archaeognatha	Meinertellidae		sp.1	X		E
Insecta	Coleoptera	Dytiscidae		sp.20	X		E
Insecta	Coleoptera	Nitidulidae		sp.1		X	E
Insecta	Coleoptera	Scydmaenidae		sp.1	X		E
Insecta	Diptera	Cecidomyiidae	Cecidomyiinae	sp.	X	X	E
Insecta	Diptera	Chironomidae		sp.	X		E
Insecta	Diptera	Chloropidae		sp.	X		E
Insecta	Diptera	Dolichopodidae		sp.	X		E
Insecta	Diptera	Psychodidae	Phlebotominae	sp.	X	X	E
Insecta	Heteroptera	Dipsocoroidea		sp.	X		E
Insecta	Heteroptera	Veliidae	*Paravelia*	jovem		X	E
Insecta	Homoptera	Cixiidae		sp.1		X	E
Insecta	Homoptera	Cixiidae		sp.8	X		E
Insecta	Hymenoptera	Formicidae	*Camponotus*	sp.1	X		E
Insecta	Hymenoptera	Formicidae	*Crematogaster*	sp.1	X		E
Insecta	Hymenoptera	Formicidae	*Eurhopalothrix*	sp.1	X		E
Insecta	Hymenoptera	Formicidae	*Hypoponera*	sp.2	X		E
Insecta	Hymenoptera	Formicidae	*Labidus*	sp.1		X	E
Insecta	Hymenoptera	Formicidae	Myrmicinae	sp.1	X		E
Insecta	Hymenoptera	Formicidae	*Pachycondyla*	sp.2	X	X	E
Insecta	Hymenoptera	Formicidae	*Pheidole*	sp.3		X	E
Insecta	Hymenoptera	Formicidae	*Solenopsis*	sp.1		X	E
Insecta	Isoptera	Termitidae	*Nasutitermes*	sp.	X	X	E
Insecta	Lepidoptera	Noctuoidea		sp.3		3	E
Insecta	Lepidoptera	Noctuoidea		sp.5		1	E
Insecta	Orthoptera	Phalangopsidae	*Paraclodes*	sp.	8		E
Insecta	Orthoptera	Phalangopsidae	*Phalangopsis*	sp.		2	E
Insecta	Psocoptera	Epipsocidae	*Mesepipsocus*	sp.1	X	X	E
Insecta	Thysanura	Nicoletiidae		sp.1	X		E
Gastropoda	Pulmonata	Systrophiidae	*Happia*	sp.	X		E
Malacostraca	Isopoda	Armadillidae		sp.1		X	E
Malacostraca	Isopoda	Dubioniscidae		sp.1		X	E
Amphibia	Anura			sp.	1		E
Mammalia	Chiroptera	Emballonuridae	*Peropteryx*	*kappleri*	1		E
